# Evaluation of new Corvis ST parameters in normal, Post-LASIK, Post-LASIK keratectasia and keratoconus eyes

**DOI:** 10.1038/s41598-020-62825-y

**Published:** 2020-03-30

**Authors:** Kaili Yang, Liyan Xu, Qi Fan, Yuwei Gu, Peng Song, Bo Zhang, Dongqing Zhao, Chenjiu Pang, Shengwei Ren

**Affiliations:** Henan Provincial People’s Hospital, Henan Eye Hospital, Henan Eye Institute, People’s Hospital of Zhengzhou University, Henan University People’s Hospital, Zhengzhou, 450003 China

**Keywords:** Corneal diseases, Refractive errors

## Abstract

The aim of this study was to evaluate the distribution of new Corneal Visualisation Scheimpflug Technology (Corvis ST) parameters in normal, Post-laser *in situ* keratomileusis (LASIK), Post-LASIK keratectasia (KE) and keratoconus (KC) eyes, and explore the diagnostic ability of these parameters in distinguishing KE from LASIK eyes. Twenty-three normal eyes, 23 LASIK eyes, 23 KE eyes and 23 KC eyes were recruited in this study. The following new Corvis ST parameters were measured: Max Inverse Radius, deformation amplitude (DA) Ratio Max [2 mm], Pachy Slope, DA Ratio Max [1 mm], Ambrosio’s relational thickness horizontal (ARTh), Integrated Radius, stiffness parameter at first applanation (SP-A1) and Corvis biomechanical index (CBI). The general linear model, linear regression model, relation analysis and receiver operating characteristic (ROC) curve were performed. The Max Inverse Radius, DA Ratio Max [2 mm], Pachy Slope, DA Ratio Max [1 mm], Integrated Radius and CBI in LASIK eyes, KE eyes and KC eyes were higher than in normal eyes, while the ARTh and SP-A1 parameters were lower than in normal eyes. The KE eyes had higher Max Inverse Radius, DA Ratio Max [2 mm], Pachy Slope, DA Ratio Max [1 mm], Integrated Radius, and lower SP-A1 value than LASIK eyes (all *P* < 0.05). The central corneal thickness was related to the Pachy Slope (r = −0.485), ARTh (r = −0.766), SP-A1 (r = 0.618) in KE eyes (all *P* < 0.05). The area under the ROC curve of Integrated Radius, Max Inverse Radius, DA Ratio Max [2 mm] and SP-A1 were above 0.800 in identifying KE from LASIK eyes. Thus, the new Corvis ST parameters were different between LASIK and KE eyes, suggesting that they might be helpful in distinguishing KE eyes from LASIK eyes. However, a further multi-center and large sample study is necessary to confirm these findings.

## Introduction

Laser *in situ* keratomileusis (LASIK), a common corneal refractive surgery, is performed to reduce spherical and cylindrical refractive errors^1^. Post-LASIK keratectasia (KE) is relatively rare, but when it occurs, it is usually considered as a serious complication of refractive surgery^2^. It was firstly reported by Seiler in 1998, and gradually generated substantial scientific interest when the number of patients who chose to undergo LASIK increased^3^. Several studies reported that the incidence of KE approximately ranged from 0.04% to 0.6% in different populations^4–6^. KE is a serious complication with visual morbidity, and the visual consequence in some instances is severe and requires corneal transplantation^7,8^.

Previous study reported that the topographic changes in KE eyes are initially subtle, then they progress accompanied by focal steepening over time^2^. Several studies summarized the disruption of biomechanical integrity in KE eyes^9–12^, and introduced the corneal biomechanical assessments measured by Ocular Response Analyzer (ORA)^13,14^. The Corneal Visualisation Scheimpflug Technology (Corvis ST) is a relatively new instrument used to estimate in more details of the corneal biomechanical properties^15^. However, evidence on the evaluation of the Corvis ST parameters in KE eyes is limited. A recent study showed that the radius and Deflection Amplitude (DLA) were significantly different among normal, LASIK, KE, and KC eyes^16^. With the development of software, new parameters such as Max Inverse Radius, deformation amplitude (DA) Ratio Max [2 mm], Pachy Slope, DA Ratio Max [1 mm], Ambrosio relational thickness horizontal (ARTh), Integrated Radius, stiffness parameter at first applanation (SP-A1) and Corvis biomechanical index (CBI) were introduced, showing to play important roles in clinical application^17,18^. However, the difference of new Corvis ST parameters among LASIK, KE and KC eyes is not reported yet. In addition, previous studies reported that basic characteristics such as age, mean keratometry (Km), intraocular pressure (IOP), and central corneal thickness (CCT) were associated with the corneal biomechanics in normal and KC eyes^19,20^. The Max Inverse Radius, SP-A1 and CBI parameters have the potential to differentiate KC eyes from normal eyes^18,21^. However, the potential ability of these new parameters in differentiating KE eyes and LASIK eyes still need to be assessed.

Thus, this study aimed to explore the distribution of new Corvis ST parameters in normal, LASIK, KE and KC eyes, to analyze the correlation between new parameters and basic characteristics in terms of age, Km, IOP and CCT. The diagnostic ability of the new Corvis ST parameters to differentiate KE eyes and LASIK eyes was also evaluated.

## Methods

### Study subjects

The study was performed at the Henan Eye Hospital & Henan Eye Institute between August 2018 and November 2019. The inclusion criteria for KC eyes were the following: based on asymmetric bowtie pattern with or without skewed axes revealed by corneal topography or KC sign detected by slit-lamp examination, such as localized stromal thinning, conical protrusion, Vogt’s striae, Fleischer’s ring or anterior stromal scar^22^. The inclusion criteria for KE eyes were the following: (1) underwent LASIK for myopia and myopic astigmatism; 2) developed KE with the same diagnosis as KC eyes. The severity of the KC eyes and KE eyes were classified according to the Amsler-Krumeich Classifcation^22^ (KC: Stage I 11, II 3, III 8, IV 1. KE: Stage I 11, II 5, III 3, IV 4). The inclusion criteria for LASIK eyes were the following: (1) underwent LASIK for myopia and myopic astigmatism at least 1 year; 2) value of the Best Corrected Visual Acuity (BCVA) LogMAR ≤ 0.1, and no presence of corneal ectasia on topographic maps. The IOP and CCT were matched among the LASIK, KE and KC eyes in order to control for possible confounding factors and better compare the differences in biomechanical parameters in different groups. The inclusion criteria for normal eyes were the following: (1) The BCVA(LogMAR) ≤ 0.1 with normal topographic maps; (2) Absence of pathologies and no ocular surgery. The exclusion criteria were the following: eyes with previous ocular surgery (except LASIK), rigid contact lens used in the last 4 weeks, soft contact lens used in the last 2 weeks, stromal scar, serious diabetes, a history of ocular disease other than KC. The statistical efficacy containing 23 cases in each group was above 0.90 measured by PASS Software, which might indicate that the study was reliable and representative to some extent.

### Examinations

The measurements were performed between 9:00 AM and 5:00 PM by the same experienced operator. The BCVA and slit lamp examination were performed by a professional ophthalmologist. The value of steep keratometry (Ks), flat keratometry (Kf), and Km were detected using the Visante Omni anterior segment OCT (Carl Zeiss Jena GmBH, Germany).

The Corvis ST (Oculus 72100, Wetzlar, Germany) instrument took Scheimpflug images of the anterior segment at a rate of 4330 frames/s, and the acceptable repeatability of Corvis ST parameters in normal and KC eyes was performed as previously described^23,24^. The corneal biomechanical response contains three phases: first applanation, highest concavity and second applanation^15,25^. The following parameters were measured by the Corvis ST Software and recorded: applanation times (from the start of air puff until the applanation^26^), corneal velocity (the speed of the corneal apex as it moves inward and outward after the time of highest concavity), DA (describing the movement of the corneal apex in vertical direction over time and is calculated as the sum of the deflection amplitude and whole eye movement), deflection length (DLL), deflection area (DLAr) and delta arc length (dArcL). In addition, the following parameters were evaluated, such as the IOP, biomechanical corrected IOP (bIOP), CCT, peak distance (PD, the distance between the two peaks of the cornea at the highest points of the cornea nasally and temporally to its center), radius of the curvature calculated during the concave phase of the deformation response^25^, and the whole eye movement during the examination. Using the updated software (software number: 1.5r1902), new Corvis ST parameters were added, such as the ratio between the deformation amplitude at the apex and at 1 or 2 mm^27^ (DA Ratio Max [1 mm], DA Ratio Max [2 mm]), Pachy Slope (reflecting the difference in the cornea thickness from the center to the periphery), Max Inverse Radius (1/radius of curvature), Integrated Radius (the integrated area under the radius of the inversed curvature during the concave phase), ARTh (defined as the ratio between the thickness at the thinnest point and the progression index that describes the thickness increase from the thinnest point to the periphery^18^), SP-A1 (calculated as the adjusted pressure at the first applanation minus IOP divided by the deflection amplitude at the first applanation^28^), and a combination parameter named CBI which was calculated based on a logistic regression formula calculated from different Corvis ST parameters^18^. All participants receiving three repeated measurements and examinations with good quality scores were included in the current analysis.

### Statistical analysis

The Kolmogorov-Smimov test was used to assess the normality of the continuous variables, and mean ± standard deviation (SD) or median (inter-quartile, IQ) was calculated to describe the values. The general linear model and the linear regression model with new Corvis ST parameters as dependent variables were applied to compare each variable among normal, LASIK, KE and KC eyes, and then pairwise comparison was performed by the Tukey–HSD (Honestly Significant Difference) method. The relationship of new Corvis ST parameters with age, Km, IOP and CCT was evaluated using the Pearson or Spearman rank correlation analysis. In addition, the receiver operating characteristic (ROC) curve was used to evaluate the diagnostic ability of the new Corvis ST parameters in distinguishing KE eyes from LASIK eyes. The statistical analysis of the current data was performed using SPSS 23.0 software package and MedCalc software. *P* < 0.05 (two-tailed) was considered statistically significant.

### Ethics approval and informed consent

This study was conducted according to the Declaration of Helsinki guidelines and all procedures involving human subjects were approved by the Institutional Review Board of Henan Eye Hospital [ethical approval number: HNEECKY-2019 (5)]. Written informed consent was obtained from all patients.

## Results

### Demographic data of the participants

Table 1 shows the demographic data among the normal, LASIK, KE and KC groups. The BCVA (LogMAR), Ks, Kf, Km, IOP and CCT were significantly different among the four groups (all *P* < 0.001), while the age was not significantly different (*P* = 0.972). The IOP and CCT were not significant different among LASIK, KE and KC eyes (all *P* > 0.05).

**Table 1:** Demographic data of participants.

Parameters(mean ± SD)	Normal (N = 23)	LASIK (N = 23)	KE (N = 23)	KC (N = 23)	*P*	P*	P**	P***	*P*^*#*^	*P*^*##*^	*P*^*@*^
Age (years)	28.91 ± 3.94	29.61 ± 7.83	29.65 ± 6.41	29.78 ± 7.89	0.972	0.985	0.982	0.971	1.000	1.000	1.000
BCVA (LogMAR)	0.00 ± 0.00	0.00 ± 0.00	0.35 ± 0.27	0.50 ± 0.53	<0.001	1.000	0.002	<0.001	0.002	<0.001	0.393
Ks (D)	43.61 ± 1.6	39.09 ± 2.65	48.18 ± 5.37	52.05 ± 5.29	<0.001	0.002	0.001	<0.001	<0.001	<0.001	0.010
Kf (D)	42.46 ± 1.26	38.22 ± 2.5	45.41 ± 4.25	48.65 ± 4.87	<0.001	0.001	0.028	<0.001	<0.001	<0.001	0.013
Km (D)	43.03 ± 1.39	38.66 ± 2.56	46.79 ± 4.75	50.38 ± 5.04	<0.001	0.001	0.006	<0.001	<0.001	<0.001	0.009
IOP(mmHg)	13.74 ± 0.65	11.43 ± 1.96	11.76 ± 2.08	11.54 ± 2.04	<0.001	<0.001	0.002	<0.001	0.925	0.997	0.976
CCT(µm)	537.04 ± 19.28	431.3 ± 30.45	427.57 ± 39.07	432.26 ± 28.15	<0.001	<0.001	<0.001	<0.001	0.975	1.000	0.952

P, general linear model; P*, Normal vs LASIK; P**, Normal vs KE; P***, Normal vs KC; P^#^, LASIK vs KE; P^##^, LASIK vs KC; P^@^, KE vs KC, Tukey-HSD method.

BCVA, best corrected visual acuity; CCT, central corneal thickness; IOP, intraocular pressure; KC, keratoconus; KE, Post-LASIK keratectasia; Kf, flat keratometry; Km, mean keratometry; Ks, steep keratometry; LASIK, laser *in situ* keratomileusis.

### Distribution of Corvis ST parameters

**Figure 1:**
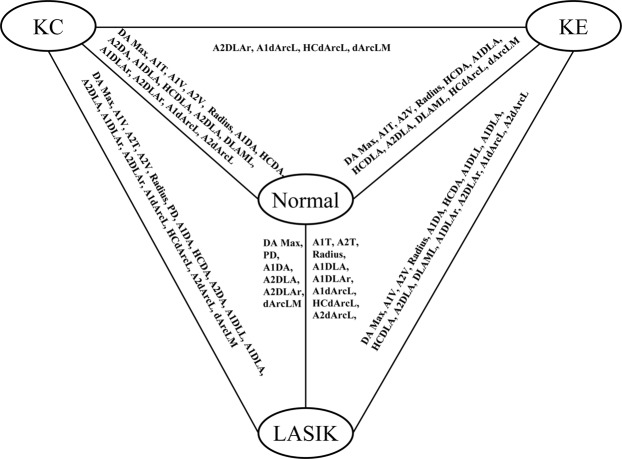
The established parameters that presented significant differences between each two groups.

The established Corvis ST parameters of the four groups are described in the Supplementary Table 1 and Fig. 1. The DA Max, A1V, A2V, Radius, A1DA, HCDA, A1DLL, deflection amplitude at the first applanation (A1DLA), HCDLA, A2DLA, max length at deflection amplitude (DLAML), A1DLAr, A2DLAr, A1dArcL, and A2dArcL were significantly different between LASIK and KE eyes (all *P* < 0.05).

**Figure 2:**
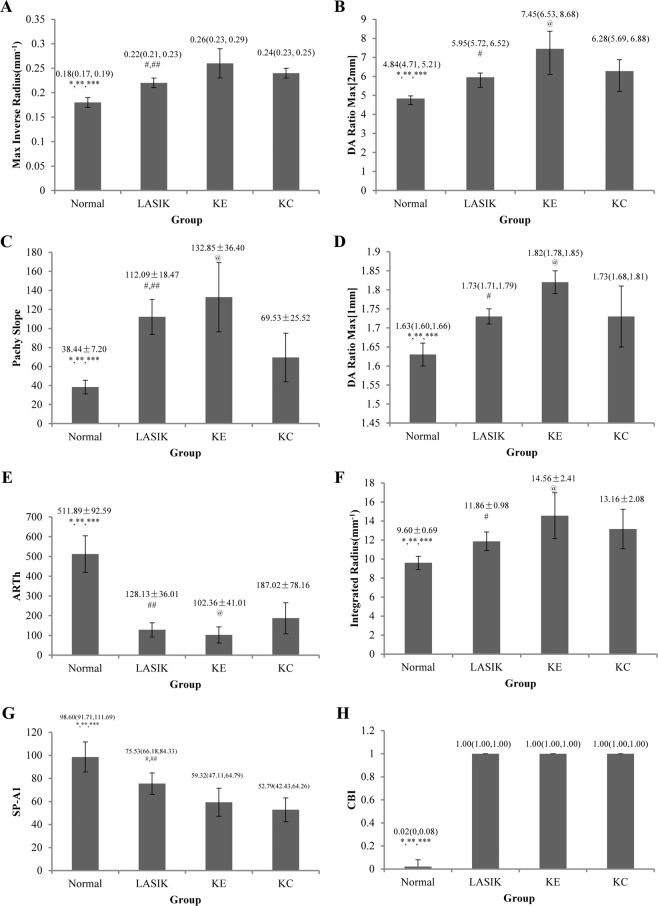
The histogram analyses for new variables measured by Corvis ST (all *P* < 0.001 for general linear model. *P < 0.05 for Normal vs LASIK; **P < 0.05 for Normal vs KE; ***P < 0.05 for Normal vs KC; ^#^*P* < 0.05 for LASIK vs KE; ^##^*P* < 0.05 for LASIK vs KC; ^@^*P* < 0.05 for KE vs KC, Tukey-HSD method). (**A**) Max Inverse Radius; (**B**) DA Ratio Max [2 mm]; (**C**) Pachy Slope; (**D**) DA Ratio Max [1 mm]; (**E**) ARTh; (**F**) Integrated Radius; (**G**) SP-A1; (**H**) CBI.

The new Corvis ST parameters among the four groups are shown in Fig. 2 and Table 2. The Max Inverse Radius, DA Ratio Max [2 mm], Pachy Slope, DA Ratio Max [1 mm], Integrated Radius and CBI in LASIK eyes, KE eyes and KC eyes were higher than normal eyes, while the ARTh and SP-A1 were lower than normal eyes (all *P* < 0.05). The KE eyes had higher values of Max Inverse Radius, DA Ratio Max [2 mm], Pachy Slope, DA Ratio Max [1 mm] and Integrated Radius, and lower SP-A1 value than LASIK eyes (all *P* < 0.05). In addition, the KC eyes had lower values of DA Ratio Max [2 mm], Pachy Slope, DA Ratio Max [1 mm], Integrated Radius, and higher ARTh value than KE eyes (all *P* < 0.05).

**Table 2:** Association of new Corvis ST parameters with different eye groups.

Parameters	Normal (Ref)	LASIK	KE	KC
Max Inverse Radius(mm^−1^)	0	0.036 (0.021, 0.051)	0.076 (0.062, 0.091)	0.058 (0.043, 0.073)
DA Ratio Max [2 mm]	0	1.123 (0.571, 1.674)	2.527 (1.988, 3.067)	1.541 (0.996, 2.087)
Pachy Slope	0	72.917 (58.531, 87.302)	94.226 (80.15, 108.303)	31.092 (16.867, 45.317)
DA Ratio Max [1 mm]	0	0.110 (0.067, 0.154)	0.178 (0.135, 0.22)	0.121 (0.078, 0.164)
ARTh	0	−382.214 (−421.535, −342.893)	−409.86 (−448.335, −371.386)	−324.865 (−363.747, −285.984)
Integrated Radius(mm^−1^)	0	2.212 (1.202, 3.221)	4.907 (3.920, 5.894)	3.560 (2.563, 4.558)
SP-A1	0	−27.459 (−35.409, −19.509)	−44.347 (−52.126, −36.568)	−47.000 (−54.861, −39.139)
CBI	0	0.948 (0.921,0.975)	0.948 (0.922, 0.974)	0.936 (0.909, 0.962)

ARTh, Ambrosio’s relational thickness horizontal; CBI, Corvis biomechanical index; DA Ratio Max [2 mm], deformation amplitude Ratio Max [2 mm]; DA Ratio Max [1 mm], deformation amplitude Ratio Max [1 mm]; SP-A1, stiffness parameter at first applanation.

### Relationship between new Corvis ST parameters and basic characteristics

**Figure 3:**
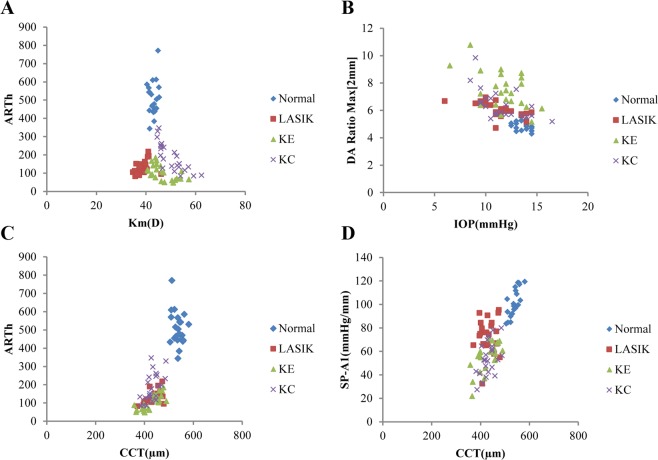
Scatter plots line graph of new Corvis ST parameters among normal, LASIK, KE and KC eyes. (**A**) ARTh vs Km; (**B**) DA Ratio Max [2 mm] vs IOP; (**C**) ARTh vs CCT; (**D**) SP-A1 vs CCT.

The coefficients between age, Km, IOP, CCT and new Corvis ST parameters are summarized in supplementary Table 2. Age was statistically and negatively correlated with Pachy Slope in normal eyes(r = −0.483) and KC eyes (r = −0.470), and positively related with SP-A1 in LASIK eyes (r = 0.489) and KC eyes (r = 0.502). Km was statistically and positively associated with ARTh in LASIK eyes (r = 0.466), while a negative correlation was found in KE eyes (r = −0.557) and KC eyes (r = −0.840, Fig. 3A). The coefficients between IOP and DA Ratio Max [2 mm] were −0.692, −0.432, −0.660 in LASIK, KE and KC groups, respectively (all *P* < 0.05, Fig. 3B). The coefficients between CCT and ARTh were 0.644, −0.776, 0.574 in LASIK, KE and KC groups, respectively (all *P* < 0.05, Fig. 3C). Similarly, the coefficients between CCT and SP-A1 were 0.071, 0.618, 0.464 in LASIK, KE and KC groups, respectively (*P*_LASIK_ = 0.747, *P*_KE_ = 0.002, *P*_KC_ = 0.026, Fig. 3D).

### ROC curve analysis of new Corvis ST parameters

The ROC results to distinguish KE eyes from LASIK eyes are shown in Table 3. The Integrated Radius had the highest predictive accuracy in distinguishing KE eyes from LASIK eyes (Sensitivity: 86.96%, Specificity: 82.61%). In addition, the area under the ROC curve of Max Inverse Radius, DA Ratio Max [2 mm] and SP-A1 was above 0.800 for all these parameters (all *P* < 0.05), while the CBI and Pachy Slope could not distinguish KE eyes from LASIK eyes (*P* > 0.05).

**Table 3:** ROC values of new Corvis ST parameters in identifying KE eyes from LASIK eyes.

Parameter	AUC(95 %*CI*)	*P*	Youden index	Cut off	Sensitivity (%)	Specificity (%)
Max Inverse Radius	0.850 (0.714, 0.938)	<0.001	0.565	>0.227	82.61	73.91
DA Ratio Max [2 mm]	0.847 (0.710, 0.936)	<0.001	0.609	>6.733	65.22	95.65
Pachy Slope	0.664 (0.509, 0.796)	0.055	0.435	>128.391	56.52	86.96
DA Ratio Max [1 mm]	0.750 (0.601, 0.866)	0.001	0.565	>1.779	78.26	78.26
ARTh	0.686 (0.533, 0.815)	0.021	0.391	≤76.134	39.13	100.00
Integrated Radius	0.885 (0.756, 0.960)	<0.001	0.696	>12.473	86.96	82.61
SP-A1	0.851 (0.715, 0.938)	<0.001	0.652	≤62.422	78.26	86.96
CBI	0.543 (0.390, 0.691)	0.148	0.087	>0.999	100.00	8.70

ARTh, Ambrosio’s relational thickness horizontal; AUC(95 %CI): area under the ROC curve (95 % Confidence interval); CBI, Corvis biomechanical index; DA Ratio Max [2 mm], deformation amplitude Ratio Max [2 mm]; DA Ratio Max [1 mm], deformation amplitude Ratio Max [1 mm]; SP-A1, stiffness parameter at first applanation.

## Discussion

It is known that KE is one of the most serious complications of LASIK^29^. This study found that KE eyes had higher values of Max Inverse Radius, DA Ratio Max [2 mm], Pachy Slope, DA Ratio Max [1 mm], Integrated Radius, and lower SP-A1 value than LASIK eyes. The values of new Corvis ST parameters were associated with age, Km, IOP and CCT. In addition, the ROC results indicated that the Max Inverse Radius, Integrated Radius and SP-A1 were helpful in distinguishing KE eyes from LASIK eyes.

KE is characterized by thinning and bulging of the cornea, leading to a myopic shift and irregular astigmatism, and reducing uncorrected and corrected visual acuity^13,30^. Earlier studies focusing on changes in topographic maps have been reported^2,13,16^. Wolle MA *et al*.^2^ summarized the topographic changes in the KE eyes, which were initially subtle, might be accompanied by focal steepening, and could be indistinguishable from KC progress over time. Ryotaro *et al*.^16^ reported that the CCT in LASIK, KE and KC eyes were not different, but it was thinner in the three groups compared to that in the normal eyes. The phenomenon of CCT reduction might be caused by tissue removal, and softening of the tissue is expected because of the structural alteration due to severing tension-bearing lamellae^31^. In addition, the current study indicated that KE eyes had higher keratometry readings, weaker sight, lower IOP and CCT values than normal eyes. The results were consistent with Ambrósio R Jr *et al*. findings, in which the operated eye with progressive corneal ectasia was compared with the unoperated fellow eye^13^.

Despite the evolution of corneal shape analysis, biomechanical understanding is of paramount importance for increasing the sensitivity in identifying cases with mild disease and characterizing the susceptibility for ectasia progression^32^. In fact, a consensus exists regarding the pathophysiology of corneal ectasia, which is considered as related to altered corneal biomechanics^33^. Furthermore, Luz A and coworkers summarized previous literatures on corneal biomechanics, and predicted corneal biomechanical assessments would have a bright future for ectasia detection^34^. As Corvis ST came into use, the consistent air puff maximal pressure was measured in every examination and more detailed information of corneal deformation could be evaluated^17^. Recent reviews summarized the practical application of Corvis ST parameters in normal eyes, KC eyes, and KE eyes^15,17^. However, the studies in terms of the comparisons among LASIK eyes, KE eyes and KC eyes are still limited. Only one study performed by Ryotaro *et al*.^16^ found that the radius, HCDA, A1DLL, A1DLA, and HCDLA were different between LASIK eyes and KE eyes and no significant difference was found between KE and KC eyes. Besides the above parameters, we also found that the DA Max, A1V, A2V, A1DA, A1DLA, A2DLA, DLAML, A1DLAr, A2DLAr, A1dArcL, A2dArcL were significantly different between LASIK and KE eyes, and the A2DLAr, A1dArcL, HCdArcL, dArcLM parameters were significant different between KE and KC eyes. Among the established parameters, the values between KE and KC eyes were similar, which is consistent with a previous study^16^. The differences of the established parameters over studies might be caused by the diversity of participant’s ethnicity, the inconsistency of the sample size and the parameters mainly concerned. An additional large sample study and multi-center study are needed to further elucidate the differences of Corvis ST parameters.

With the updated versions of Corvis ST software, new parameters were added for a better assessment of the biomechanical parameters^17^. Several studies found that the new parameters had the potential ability to distinguish KC eyes from normal eyes^18^. The current study found that the Max Inverse Radius, DA Ratio Max [2 mm], Pachy Slope, DA Ratio Max [1 mm], Integrated Radius and CBI in LASIK eyes, KE eyes and KC eyes were higher than that in normal eyes, while the ARTh and SP-A1 were lower than that in normal eyes. The results indicated that the normal eyes had stiffer corneas than LASIK, KE and KC eyes^25^. In addition, this study found that the Max Inverse Radius values in KE and KC eyes were higher than that in LASIK, which means that the LASIK eyes might have stiffer corneas than KE and KC eyes. The DA Ratio Max [1 mm] or DA Ratio Max [2 mm] increases more in softer corneas than in stiffer corneas^18^. The current study found that the DA Ratio Max [1 mm], Pachy Slope, DA Ratio Max [2 mm], and Integrated Radius values in KE eyes were significantly higher than that in LASIK eyes and KC eyes, and the SP-A1 in KE eyes were significantly lower than that in LASIK eyes. Furthermore, the ROC results showed that the Max Inverse Radius, DA Ratio Max [2 mm], Integrated Radius and SP-A1 had acceptable values, which could be helpful in distinguishing KE eyes from LASIK eyes. This study also found that KC eyes had lower values of DA Ratio Max [2 mm], Pachy Slope, DA Ratio Max [1 mm], Integrated Radius, and higher ARTh value than KE eyes, which might provide evidence that KE eyes had softer corneal tissue than KC eyes. Furthermore, the values of Max Inverse Radius, Pachy Slope, ARTh and SP-A1 were different between LASIK eyes and KC eyes, which could be paid more attention when investigating the LASIK and KC eyes in clinical application. The value of CBI is based on a logistic regression formula calculated from different Corvis ST parameters (A1V, ARTh, SP-A1, DA Ratio Max [2 mm], DA Ratio Max [1 mm], and DLA). A study reported that values below 0.25 indicate a low risk of developing ectasia, values between 0.25 and 0.5 indicate a moderate risk of developing ectasia, and values above 0.5 indicate a high risk of developing ectasia^18^. The CBI values among LASIK, KE and KC eyes were not significantly different, which indicated that the value could not be used to differentiate LASIK, KE and KC eyes. The Corvis ST provides parameters to reflect the corneal biomechanical characteristics, and the differences of Max Inverse Radius, DA Ratio Max [2 mm], Pachy Slope, DA Ratio Max [1 mm], and Integrated Radius might help us to understand the corneal biomechanics of LASIK, KE and KC eyes. Whether these differences could be extended to other studies remained to be further evaluated, and more research is needed.

This study showed that the age, Km, IOP and CCT were statistically correlated with the new Corvis ST parameters in normal and KC eyes, which was consistent with previous studies^19,20^. However, the association between the basic parameters and new Corvis ST parameters in LASIK and KE eyes is not reported. It was reported that the corneal tissue between 30 and 90 years clearly exhibited stiffening with age^35^. This study found that the age was only positively associated with SP-A1 in LASIK eyes, while no relationship was found in KE eyes. The Km is an important parameter of the ocular optical system, and represents the optical refractive power of the eye^36^. As an important parameter to evaluate the corneal ectasia, Km was found significantly associated with ARTh, CBI in LASIK eyes, and with Pachy Slope, CBI in KE eyes. The change of IOP is an indicator of visual impairments, and several studies reported that IOP is associated with Corvis ST parameters^15,18^. IOP in the current study was found related with DA Ratio Max [2 mm], DA Ratio Max [1 mm], and Integrated Radius in KE eyes. Besides that, this study found that CCT was related to Pachy Slope, ARTh and SP-A1 in KE eyes. The CCT is reduced with the tissue removal in refractive surgery^31^. The thinning of the cornea in KC patients decreases the biomechanical properties, which in turn result in focal weakening of the cornea, and the decrease of the corneal properties may further thin the cornea^11,37^. Further multi-center study should be conducted to explore the association between new Corvis ST parameters and the subject’s basic characteristics.

This study evaluated the difference of new Corvis ST parameters among normal, LASIK, KE, and KC eyes. However, some limitations should be mentioned. Firstly, the current study recruited 23 eyes in each group, which is a relatively small number. Considering that the Corvis ST Software was introduced in recent years, and the prevalence of KE is relatively low, the results could provide some evidences in evaluating the corneal biomechanics. Further multi-center and large sample study would be performed in the near future. Secondly, this study lacks the preoperative corneal biomechanical information, and did not evaluate the changes of corneal biomechanics over time. A prospective clinical study is ongoing to investigate the corneal biomechanics of LASIK, KE and KC eyes in different follow-up times.

In conclusion, the new Corvis ST parameters were different between LASIK and KE eyes, and might help to distinguish LASIK eyes from KE eyes. However, the effect of age, Km, IOP and CCT on these parameters should be considered in clinical application. Additional studies are required to explore the characteristics of corneal biomechanics in other population to extend the clinical application.

### Data statement

All relevant data are included in the papers and its Supporting Information files. Contact to Dr. Shengwei Ren (shengweiren1984@163.com) for additional information regarding data access.

## Supplementary information

Supplementary information.
