# Supplementary material for: Evaluation of new Corvis ST parameters in normal, Post-LASIK, Post-LASIK keratectasia and keratoconus eyes
Source: Sci Rep. 2020 Mar 30;10:5676. doi: 10.1038/s41598-020-62825-y (PMC7105482; doi:10.1038/s41598-020-62825-y)
Supplement: Supplementary file 1 — Supplementary information. [file 41598_2020_62825_MOESM1_ESM.pdf]

Evaluation of new Corvis ST parameters in normal, Post-LASIK, Post-LASIK keratectasia and keratoconus eyes

Authors:

Kaili Yang, Liyan Xu, Qi Fan, Yuwei Gu, Peng Song, Bo Zhang, Dongqing Zhao, Chenjiu Pang*, Shengwei Ren*

Authors affiliations:

Henan Provincial People’s Hospital, Henan Eye Hospital, Henan Eye Institute, People's Hospital of Zhengzhou University, Henan University People's Hospital, Zhengzhou, 450003, China.

*** Correspondence authors**

Shengwei Ren and Chenjiu Pang

Henan Provincial People’s Hospital

Henan Eye Hospital, Henan Eye Institute

People's Hospital of Zhengzhou University

Henan University People's Hospital

7 Weiwu Road, Zhengzhou, 450003, Henan, *PR* China

Phone: +86 371 65580908

E-mail: shengweiren1984@163.com and pangcj999@163.com

Supplementary Table 1 Data of Corvis ST parameters among normal, LASIK, KE and KC eyes.

Parameters(mean±SD)	Normal	LASIK	KE	KC	P	P*	P**	P***	$P^{\#}$	$P^{\#\#}$	$P^{@}$
DA Max(mm)	1.15±0.07	1.22±0.09	1.32±0.13	1.32±0.12	<0.001	0.078	<0.001	<0.001	0.009	0.010	1.000
A1T(ms)	6.97±0.12	6.77±0.18	6.77±0.26	6.65±0.22	<0.001	0.006	0.007	<0.001	1.000	0.187	0.17
A1V(m/s)	0.16±0.01	0.15±0.02	0.17±0.03	0.18±0.01	<0.001	0.567	0.117	0.012	0.003	<0.0010	0.813
A2T(ms)	22.3±0.28	22.61±0.37	22.56±0.26	22.30±0.43	0.002	0.015	0.057	1.000	0.961	0.015	0.056
A2V(m/s)	-0.29±0.03	-0.30±0.03	-0.34±0.05	-0.33±0.06	<0.001	0.989	0.003	0.012	0.009	0.028	0.977
HCT(ms)	17.38±0.47	17.53±0.73	17.38±0.53	17.49±0.55	0.746	0.813	1.000	0.916	0.813	0.995	0.916
PD(mm)	5.21±0.22	5.43±0.17	5.30±0.29	5.14±0.31	0.001	0.020	0.602	0.78	0.322	0.001	0.134
Radius(mm)	6.50±0.38	5.70±0.46	4.73±0.64	5.05±0.66	<0.001	<0.001	<0.001	<0.001	<0.001	0.001	0.208
A1DA(mm)	0.12±0.01	0.11±0.01	0.13±0.02	0.14±0.01	<0.001	<0.001	0.055	0.001	<0.001	<0.001	0.492
HCDA(mm)	1.15±0.07	1.22±0.09	1.32±0.13	1.32±0.12	<0.001	0.078	<0.001	<0.001	0.009	0.01	1.000
A2DA(mm)	0.37±0.06	0.37±0.07	0.39±0.08	0.44±0.10	0.012	0.983	0.945	0.036	0.794	0.013	0.134
A1DLL(mm)	2.10±0.43	1.92±0.30	2.23±0.24	2.29±0.16	<0.001	0.176	0.492	0.156	0.005	<0.001	0.915
HCDLL(mm)	6.61±0.54	6.40±0.86	6.00±1.04	6.38±0.50	0.074	0.78	0.047	0.732	0.321	1.000	0.365
A2DLL(mm)	2.79±1.05	2.91±1.18	2.99±1.06	3.19±0.80	0.612	0.977	0.918	0.555	0.995	0.808	0.917
A1DLA(mm)	0.09±0.01	0.07±0.01	0.10±0.02	0.10±0.01	<0.001	0.002	0.001	<0.001	<0.001	<0.001	1.000
HCDLA(mm)	0.98±0.07	1.04±0.09	1.15±0.13	1.11±0.15	<0.001	0.36	<0.001	0.002	0.009	0.176	0.629
A2DLA(mm)	0.10±0.01	0.08±0.02	0.12±0.02	0.12±0.03	<0.001	0.005	0.026	0.001	<0.001	<0.001	0.616
DLAML(mm)	0.99±0.07	1.06±0.09	1.16±0.13	1.13±0.15	<0.001	0.182	<0.001	<0.001	0.012	0.14	0.763
DLAMT(ms)	16.54±0.63	16.13±0.82	16.26±0.87	16.09±0.87	0.228	0.321	0.659	0.234	0.942	0.998	0.876
WEMA(mm)	0.28±0.06	0.29±0.07	0.28±0.08	0.32±0.10	0.183	0.934	1.000	0.224	0.934	0.544	0.224
WEMT(ms)	22.26±0.67	22.25±1.07	22.58±0.71	22.04±0.67	0.154	1.000	0.528	0.787	0.488	0.82	0.107
A1DLAr(mm^2)	0.15±0.02	0.12±0.04	0.17±0.05	0.19±0.04	<0.001	0.013	0.502	0.004	<0.001	<0.001	0.169
HCDLAr(mm^2)	3.64±0.44	3.91±0.50	4.08±0.64	3.90±0.74	0.096	0.415	0.062	0.451	0.757	1.000	0.722
A2DLAr(mm^2)	0.2±0.03	0.14±0.04	0.23±0.08	0.29±0.10	<0.001	0.02	0.459	0.001	<0.001	<0.001	0.048
A1dArcL(mm)	-0.01±0.00	-0.01±0.00	-0.02±0.00	-0.02±0.00	<0.001	0.002	0.783	0.002	<0.001	<0.001	0.027
HCdArcL(mm)	-0.13±0.02	-0.06±0.04	-0.07±0.04	-0.11±0.04	<0.001	<0.001	<0.001	0.433	0.606	<0.001	0.01
A2dArcL(mm)	-0.02±0.00	-0.01±0.01	-0.02±0.01	-0.03±0.01	<0.001	0.04	0.656	0.002	0.001	<0.001	0.055
dArcLM(mm)	-0.14±0.02	-0.09±0.04	-0.10±0.05	-0.14±0.04	<0.001	<0.001	0.001	0.963	0.923	0.001	0.007
bIOP(mmHg)	13.99±0.77	13.72±1.79	14.17±2.17	13.89±2.11	0.864	0.957	0.988	0.997	0.838	0.989	0.953

P, general linear model; P*, Normal *vs* LASIK; P**, Normal *vs* KE; P***, Normal *vs* KC; $P^{\#}$, LASIK *vs* KE; $P^{\#\#}$, LASIK *vs* KC; $P^{@}$, KE *vs* KC, Tukey-HSD method

Supplementary Table 2 Correlation of new Corvis ST parameters with age, Km, IOP and CCT.

Parameters/Group	Age		Km		IOP		CCT	
	r	*P*	r	*P*	r	*P*	r	*P*
**Normal**								
Max Inverse Radius	-0.188	0.389	0.408	0.053	0.045	0.840	-0.186	0.396
DA Ratio Max[2mm]	-0.085	0.701	0.752	<0.001	-0.257	0.237	-0.530	0.009
Pachy Slope	-0.483	0.019	-0.088	0.689	-0.054	0.808	0.326	0.129
DA Ratio Max[1mm]	-0.225	0.302	0.679	<0.001	-0.019	0.932	-0.261	0.229
ARTh	0.280	0.196	0.127	0.564	-0.038	0.865	-0.267	0.218
Integrated Radius	-0.148	0.501	0.305	0.156	-0.125	0.569	-0.026	0.905
SP-A1	-0.079	0.721	-0.390	0.066	0.212	0.331	0.788	<0.001
CBI	-0.254	0.242	0.203	0.320	-0.082	0.709	-0.148	0.499
**LASIK**								
Max Inverse Radius	0.112	0.610	-0.313	0.145	-0.012	0.956	-0.669	<0.001
DA Ratio Max[2mm]	-0.406	0.055	-0.214	0.326	-0.692	<0.001	-0.436	0.038
Pachy Slope	0.379	0.075	-0.021	0.923	-0.098	0.655	-0.179	0.413
DA Ratio Max[1mm]	-0.172	0.433	-0.317	0.141	-0.501	0.015	-0.593	0.003
ARTh	-0.262	0.227	0.466	0.025	0.251	0.248	0.644	0.001
Integrated Radius	0.084	0.703	-0.300	0.124	-0.302	0.162	-0.658	0.001
SP-A1	0.489	0.018	-0.104	0.637	0.775	<0.001	0.071	0.747
CBI	-0.187	0.393	-0.463	0.026	-0.176	0.422	-0.463	0.026
**KE**								
Max Inverse Radius	-0.045	0.839	0.256	0.239	-0.052	0.813	-0.376	0.077
DA Ratio Max[2mm]	0.074	0.738	0.031	0.890	-0.432	0.040	-0.264	0.224
Pachy Slope	0.186	0.394	0.688	<0.001	0.038	0.864	-0.485	0.019
DA Ratio Max[1mm]	-0.185	0.397	-0.092	0.676	-0.516	0.012	-0.214	0.327
ARTh	-0.045	0.839	-0.557	0.006	0.033	0.880	-0.766	<0.001
Integrated Radius	-0.150	0.496	0.051	0.817	-0.440	0.035	-0.348	0.104
SP-A1	0.233	0.286	-0.331	0.122	0.264	0.223	0.618	0.002
CBI	-	-	-	-	-	-	-	-
**KC**								
Max Inverse Radius	-0.505	0.014	0.482	0.020	-0.289	0.180	-0.323	0.132
DA Ratio Max[2mm]	-0.347	0.105	0.689	<0.001	-0.660	0.001	-0.348	0.103
Pachy Slope	-0.470	0.024	0.655	0.001	-0.335	0.118	-0.419	0.047
DA Ratio Max[1mm]	-0.241	0.268	0.534	0.009	-0.519	0.011	-0.272	0.209
ARTh	0.499	0.015	-0.840	<0.001	0.439	0.036	0.574	0.004
Integrated Radius	-0.442	0.035	0.496	0.016	-0.620	0.002	-0.344	0.108
SP-A1	0.502	0.015	-0.818	<0.001	0.721	<0.001	0.464	0.026
CBI	-0.598	0.003	0.769	<0.001	-0.476	0.022	-0.506	0.014

Spearman correlation: Max Inverse Radius, DA Ratio Max [2mm], DA Ratio Max [1mm], ARTh, SP-A1 and CBI. Pearson correlation: Pachy Slope and Integrated Radius.
